# Safety and efficacy profile of mogamulizumab (Poteligeo) in the treatment of cancers: an update evidence from 14 studies

**DOI:** 10.1186/s12885-021-08363-w

**Published:** 2021-05-26

**Authors:** Ting Zhang, Jing Sun, Jinying Li, Yunuo Zhao, Tao Zhang, Ruoning Yang, Xuelei Ma

**Affiliations:** 1 Department of Biotherapy, State Key Laboratory of Biotherapy, West China Hospital, Cancer Center, Sichuan University, No. 37 Guo Xue Alley, Chengdu, 610041 Sichuan China; 2 West China Hospital, West China School of Medicine, Sichuan University, Chengdu, China; 3 Qingdao central hospital, Qingdao University, Qingdao, Shandong China; 4 Department of radiotherapy, Qingdao central hospital, Qingdao University, Qingdao, Shandong China

**Keywords:** Mogamulizumab, Malignant lymphoma, Solid tumors, CCR4, Meta-analysis

## Abstract

**Background:**

CC chemokine receptor 4 (CCR4), the receptor for CCL22 and CCL17, is expressed on the surface of effector Tregs that have the highest suppressive effects on antitumor immune response. CCR4 is also widely expressed on the surface of tumor cells from patients with adult T-cell leukemia/lymphoma (ATL), peripheral T-cell lymphoma (PTCL) and cutaneous T-cell lymphoma (CTCL). Mogamulizumab is a humanized, IgG1 kappa monoclonal antibody that is directed against CCR4. By reducing the number of CCR4-positive Tregs and tumor cells, the mogamulizumab can reduce tumor burden and boost antitumor immunity to achieve antitumor effects.

**Methods:**

We examined the PubMed and ClinicalTrials.gov until 1 February 2020. Considering variability in different studies, we selected the adverse events (AEs), overall survival (OS), progression-free survival (PFS), objective responses rate (ORR) and Hazard Ratio (HR) for PFS to evaluate the safety and efficacy profile of mogamulizumab.

**Results:**

When patients were treated with mogamulizumab monotherapy, the most common all-grade AEs were lymphopenia, infusion reaction, fever, rash and chills while the most common grade ≥ 3 AEs were lymphopenia, neutropenia and rash. When patients were treated with combined therapy of mogamulizumab and other drugs, the most common all-grade AEs were neutropenia, anaemia, lymphopenia and gastrointestinal disorder, while the most common grade ≥ 3 AEs was lymphopenia. For patients treated with mogamulizumab monotherapy, the pooled ORR and mean PFS were 0.430 (95% CI: 0.393–0.469) and 1.060 months (95% CI: 1.043–1.077), respectively. For patients treated with combined therapy of mogamulizumab and other drugs, the pooled ORR was 0.203 (95% CI: 0.022–0.746) while the pooled PFS and OS were 2.093 months (95% CI: 1.602–2.584) and 6.591 months (95% CI: 6.014–7.167), respectively.

**Conclusions:**

Based on present evidence, we believed that mogamulizumab had clinically meaningful antitumor activity with acceptable toxicity which is a novel therapy in treating patients with cancers.

## Background

CC chemokine receptor 4 (CCR4) is the receptor for two CC chemokine ligands (CCLs)- CCL22 (also called macrophage-derived chemokine) and CCL17 (thymus activation-regulated chemokine) [1]. By binding with its ligands, CCR4 is implicated in lymphocyte trafficking to the skin and migration of CCR4-positive Tregs [2–4]. CCR4 is predominantly expressed on the surface of effector Tregs that have the highest suppressive effects on antitumor immune response. CCR4 is also widely expressed on surface of tumor cells of most patients with adult T-cell leukemia/lymphoma (ATL) and is selectively expressed in approximately 40% of patients with other subtypes of peripheral T-cell lymphoma (PTCL) and cutaneous T-cell lymphoma (CTCL) [5–10]. Mogamulizumab (also named as KW-0761, poteligeo) is a humanized, IgG1 kappa monoclonal antibody that is directed against CCR4 [10–12]. Previous studies have demonstrated that mogamulizumab can highly enhance antitumor effects by reducing CCR4-positive leukemic cells and inducing Tregs depletion [1]. So far, a series of phase I/II/III trials on mogamulizumab for various cancers have been completed. From these trials, we suppose that cancer patients treated with mogamulizumab can achieve significant treatment responses. We also focus on the adverse events of mogamulizumab or mogamulizumab-related therapies. However, there is no evidence-based systematic review on the safety and efficacy of mogamulizumab in treating patients with cancer. It is urgent and important to summarize those results, offering evidence-based references to direct clinical decisions. In this meta-analysis, we focus on the safety and efficacy of mogamulizumab in the treatment of various cancers based on selected clinical trials.

## Methods

### Literature search and selection

We followed the guidelines of PRISMA (Preferred Reporting Items for Systematic reviews and Meta-Analyses) to complete the meta-analysis. The trials were identified through PubMed and ClinicalTrials.gov without any language restrictions until 1 February 2020. The keywords included “KW-0761”, “mogamulizumab” and “poteligeo”. After duplicates eliminating, two authors screened the studies independently. When there were disagreements, we referred to the opinions of a third author. Full texts of selective trials were downloaded and assessed in strict accordance with the following criteria for eligibility. Additionally, we screened the references of selected trials for potentially relevant trials.

### Inclusion and excluded criteria

All eligible studies had to satisfy the following criteria: (i) the studies were clinical trials containing the efficacy or safety data of mogamulizumab or mogamulizumab-related therapies; (ii) the patients enrolled in these trials were suffering from cancers; (iii) the studies reported any of the following information: adverse events (AEs), overall survival (OS), progression-free survival (PFS), objective responses rate (ORR), and Hazard Ratio(HR) for PFS; (iiii) the studies used mogamulizumab as a single drug or in combination with other drugs. The exclusion criteria were: (i) the studies were not clinical trials; (ii) the studies lacked available data or the full texts were inaccessible.

### Data extraction

Data extraction was performed independently by two authors and disagreements were adjudicated by a third author. In this meta-analysis, we extracted basic information including first author’s name, clinical trial registration number, year, study phase, total number of patients, gender, age, treatment regime, tumor type and assessment. The AEs (all grades and grades ≥3), OS, PFS, ORR and HR for PFS were needed to assess the efficacy and safety of mogamulizumab. For safety endpoints, the data we extracted from the eligible trials were grade ≥ 3 and all-grade AEs according to Common Terminology Criteria for Adverse Events (CTCAE). For efficacy endpoints, we collected the OS and PFS directly in each included study while we collected the ORR directly or calculated based on the accessible data. The HR and 95% confidence interval (CI) for PFS were extracted following Wan’s method [13].

## Results

### Study selection

**Fig. 1:**
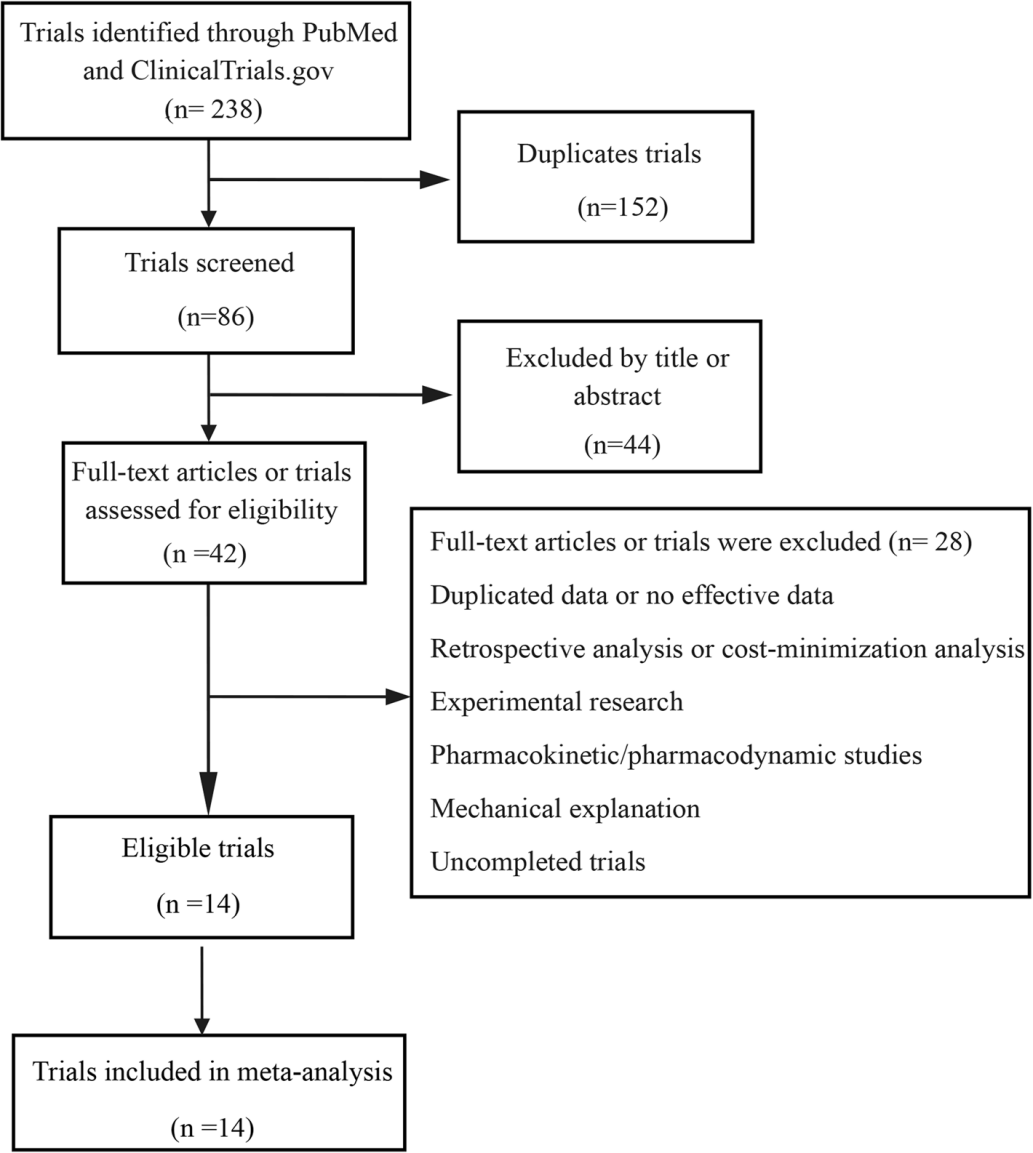
Flow diagram of literature selection for systemic reviews and meta-analyses (PRISMA)

Through a comprehensive search, we found 213 articles in PubMed and 7 trials in ClinicalTrials.gov. After duplicate, there were 73 articles and 1 trial left. By screening the titles and abstracts, we excluded 44 articles on the basis of exclusion criteria. Then we viewed the full texts of remained studies, and finally, 13 articles and 1 trial were involved according to the inclusion and exclusion criteria [14–27]. The overall filter procedures and results were shown in Fig. 1.

### Study characteristics

The basic information of eligible studies was listed on Table 1. The trials were conducted from 2010 to 2019, including 5 phase I trials, 1 phase I-II trial, 6 phase II trials, 1 phase III trial and 1 unspecified trial. Among them, patients in 2 phase I trials administered mogamulizumab intravenously at a dose of 0.01, 0.1, 0.5, 1.0 mg/kg [18, 22]. Patients in 3 phase I trials and 2 phase II trials received mogamulizumab in combination with other drugs [16, 24–27]. And the rest received mogamulizumab monotherapy at a dose of 1.0 mg/kg [14–17, 19–21, 23]. There were total 1290 patients enrolled in these studies, including patients with ATL, PTCL, CTCL, ect.

**Table 1:** Basic characteristics of the included trials

First author	Clinical trial registration number	Year	Phase	Sample size	Gender		Age	Treatment	Disease	Assessment (Response)	Assessment (AE)
					Male	Female					
Yamamoto, K.	NCT00355472	2010	I	16	8	8	62 (46–69)	mogamulizumab	relapsed CCR4^**+**^ ATL or PTCL	RECIL	CTCAE
Ishida, T.	NCT00920790	2012	II	27	12	15	64 (49–83)	mogamulizumab	relapsed CCR4^**+**^ ATL	RECIL	CTCAE
Ogura, M.	NCT01192984	2014	II	37	23	14	64 (33–80)	mogamulizumab	relapsed CCR4^**+**^ PTCL or CTCL	RECIL	CTCAE
Duvic, M.	NCT00888927	2015	I-II	41	24	17	66 (35–85)	mogamulizumab	CTCL or PTCL	RECIL	CTCAE
Ishida, T.	NCT01173887	2015	II	53	28	25	–	mLSG15 + mogamulizumab or mLSG15	aggressive ATL	RECIL	CTCAE
Kurose, K.	NCT01929486	2015	I	10	–	–	–	mogamulizumab	LC or EC	RECIST	CTCAE
Zinzani, P. L.	NCT01611142	2016	II	38	23	15	58.5 (19–87)	mogamulizumab	PTCL	RECIL	CTCAE
Ishitsuka, K.	UMIN000025368	2017	II	484	258	226	–	mogamulizumab or mogamulizumab with other drugs	ATL and others	RECIL	CTCAE
Kim, Y. H.	NCT01728805	2018	III	372	216	156	64.5 (54–73)	mogamulizumab or vorinostat	CTCL	RECIL	CTCAE
Nakashima, J.	–	2018	–	45	27	18	69 (43–89)	mogamulizumab	relapsed or refractory ATL	RECIL	CTCAE
Phillips, A. A.	NCT01626664	2018	II	34	37	71	53 (22–82)	mogamulizumab, pralatrexate, gemcitabine plus oxaliplatin or DHAP	ATL	RECIL	CTCAE
–	NCT02358473	2018	I	13	5	8	–	mogamulizumab +docetaxel	NSCLC	RECIST	CTCAE
Doi, T.	NCT02476123	2019	I	96	72	24	63 (56–68)	mogamulizumab +nivolumab	NSCLC, SCLC, GC, EC, HCC, PA	RECIST	CTCAE
Cohen, E. E. W.	NCT02444793	2019	I	24	19	5	63.9 (53–75)	mogamulizumab +utomilumab	CRC, NSCLC, OC, SCCHN	RECIST	CTCAE

*Abbreviations*: *ATL* adult T-cell leukemia-lymphoma, *PTCL* peripheral T-cell lymphoma, *CTCL* cutaneous T-cell lymphoma, *LC* lung cancer, *EC* esophageal cancer, *NSCLC* non-small cell lung cancer, *SCLC* small cell lung cancer, *GC* gastric cancer, *HCC* hepatocellular carcinoma, *PA* pancreatic adenocarcinoma, *CRC* colorectal cancer, *OC* ovarian cancer, *SCCHN* squamous cell cancer of head and neck, *mLSG15* modified LSG15 regimen (VCAP-AMP-VECP: vincristine, cyclophosphamide, doxorubicin and prednisolone; doxorubicin, ranimustine and prednisolone; vindesine, etoposide, carboplatin and prednisolone), *AE* adverse events, *RECIL* Response Evaluation Criteria in Lymphoma, *RECIST* Response Evaluation Criteria in Solid Tumors, *CTCAE* National Cancer Institute Common Terminology Criteria for Adverse Events

### Overall adverse events analysis

The safety data, grade ≥ 3 or all-grade AEs we extracted, were used to calculate the AEs rate to assess the safety of mogamulizumab. The details of the results were presented in Tables 2, 3. In all eligible trials administered mogamulizumab monotherapy, we divided these trials into low dose group (mogamulizumab≤0.1 mg/kg), medium dose group (0.5 mg/kg) and high dose group (1.0 mg/kg) in accordance with the dose. In low dose group, lymphopenia was the most common all-grade AEs and grade ≥ 3 AEs with the highest rate of 0.700 (95% CI: 0.375–0.900) and 0.401 (95% CI: 0.158–0.705), respectively. In medium dose group, the most common all-grade AEs were leukopenia and lymphopenia with the same rate of 0.875 (95% CI: 0.463–0.983) while leukopenia (0.767, 95% CI: 0.337–0.955) was the only grade ≥ 3 AEs. In high dose group, the common all-grade AEs were lymphopenia (0.805, 95% CI: 0.432–0.957), infusion reaction (0.607, 95% CI: 0.062–0.973), fever (0.472, 95% CI: 0.116–0.859), rash (0.407, 95% CI: 0.210–0.639) and chills (0.401, 95% CI: 0.129–0.751), while lymphopenia (0.648, 95% CI: 0.482–0.787) was the most common grade ≥ 3 AEs. The rest of all-grade and grade ≥ 3 AEs were happened relatively less. In the trials administered mogamulizumab in combination with other drugs, the most common all-grade AEs were neutropenia (0.812, 95% CI: 0.035–0.998), anaemia (0.687, 95% CI: 0.017–0.996), lymphopenia (0.619, 95% CI: 0.007–0.997) and gastrointestinal disorder (0.599, 95% CI: 0.001–0.999). The lymphopenia (0.568, 95% CI: 0.004–0.998) was the most common grade ≥ 3 AEs while other grade ≥ 3 AEs were relatively rare.

**Table 2:** Summary results of the all-grade and grade ≥ 3 adverse events (AEs) in mogamulizumab monotherapy

adverse events	All-grade						Grade ≥ 3					
	No. of Studies	No. of Patients	model	Event rate with 95% CI	Z value	***p*** value	No. of Studies	No. of Patients	model	Event rate with 95% CI	Z value	***p*** value
KW-0761 < =0.1 mg/kg												
Hematologic												
Lymphopenia	2	10	Fixed	0.700 (0.375–0.900)	1.224	0.221	2	10	Fixed	0.401 (0.158–0.705)	− 0.619	0.536
Nonhematologic												
Fever	2	10	Fixed	0.212 (0.052–0.569)	−1.618	0.106						
Rash	2	10	Random	0.351 (0.045–0.860)	−0.497	0.619						
KW-0761 = 0.5 mg/kg												
Hematologic												
Leukopenia	2	6	Fixed	0.875 (0.463–0.983)	1.820	0.069	2	6	Fixed	0.767 (0.337–0.955)	1.250	0.211
Lymphopenia	2	6	Fixed	0.875 (0.463–0.983)	1.820	0.069						
Neutropenia	2	6	Fixed	0.587 (0.181–0.902)	0.370	0.711						
KW-0761 = 1.0 mg/kg												
Hematologic												
Anaemia	5	316o	Random	0.097 (0.040–0.216)	−4.626	0.000	3	122	Fixed	0.056 (0.025–0.119)	−6.720	0.000
Leukopenia	5	127	Random	0.310 (0.125–0.586)	−1.368	0.171						
Lymphopenia	4	80	Random	0.805 (0.432–0.957)	1.643	0.100	3	43	Random	0.648 (0.482–0.787)	1.757	0.079
Neutropenia	6	165	Random	0.228 (0.104–0.431)	−2.543	0.011	5	155	Fixed	0.139 (0.087–0.213)	−6.915	0.000
Thrombocytopenia	4	149	Random	0.273 (0.131–0.483)	−2.108	0.035	4	149	Fixed	0.117 (0.071–0.185)	−7.311	0.000
Gastrointestinal disorders												
Diarrhea	3	90	Fixed	0.158 (0.096–0.249)	−5.743	0.000						
Vomiting	2	80	Fixed	0.128 (0.107–0.154)	−5.617	0.000						
Nausea	4	154	Random	0.147 (0.064–0.304)	−3.716	0.000	2	80	Fixed	0.039 (0.013–0.114)	− 5.436	0.000
General disorders												
Chills	2	69	Random	0.401 (0.129–0.751)	−0.522	0.602						
Fatigue	2	89	Random	0.096 (0.024–0.312)	− 3.022	0.003						
Fever	3	43	Random	0.472 (0.116–0.859)	−0.116	0.907						
Pyrexia	5	348	Random	0.139 (0.062–0.283)	−4.002	0.000						
Headache	3	127	Random	0.115 (0.046–0.259)	−4.048	0.000						
Infections and infestations												
Infection	2	84	Random	0.081 (0.015–0.335)	−2.730	0.006	2	84	Fixed	0.084 (0.038–0.175)	−5.571	0.000
Injury, poisoning and procedural complications												
Infusion reaction	2	64	Random	0.607 (0.062–0.973)	0.272	0.786						
Infusion-related reaction	4	311	Random	0.135 (0.032–0.420)	−2.370	0.018						
Metabolism and nutrition disorders /investigations												
ALT	4	121	Random	0.172 (0.063–0.391)	−2.731	0.006	3	111	Fixed	0.042 (0.016–0.107)	−6.107	0.000
AST	3	84	Random	0.167 (0.040–0.494)	−1.990	0.047	2	74	Fixed	0.049 (0.016–0.140)	−5.007	0.000
Decreased appetite	2	231	Random	0.017 (0.002–0.119)	−3.885	0.000						
CRP	2	16	Fixed	0.128 (0.032–0.395)	−2.521	0.012						
Hypercalcemia	2	37	Fixed	0.108 (0.041–0.255)	− 3.983	0.000						
Hypertension	2	33	Fixed	0.277 (0.150–0.453)	− 2.443	0.015						
Hyperuricemia	2	37	Fixed	0.137 (0.058–0.289)	−3.825	0.000						
Hypokalemia	2	64	Fixed	0.083 (0.035–0.184)	−5.133	0.000	2	64	Fixed	0.053 (0.017–0.151)	−4.857	0.000
Hypophosphatemia	2	64	Fixed	0.156 (0.086–0.267)	− 4895	0.000						
Hypotension	2	65	Fixed	0.139 (0.074–0.246)	−5.084	0.000						
Weight change	2	74	Random	0.077 (0.008–0.447)	−2.143	0.032						
Musculoskeletal and connective tissue disorders												
Polymyositis	2	221	Fixed	0.012 (0.003–0.047)	−6.179	0.000						
Arthralgia	2	222	Random	0.036 (0.004–0.278)	−2.767	0.006						
Respiratory, thoracic and mediastinal disorders												
Hypoxemia	2	33	Fixed	0.182 (0.084–0.350)	−3.330	0.001						
Pneumonia	2	221	Fixed	0.023 (0.009–0.053)	−8.316	0.000	2	75	Fixed	0.042 (0.014–0.123)	−5.293	0.000
Skin and subcutaneous tissue disorders												
Pruritus	4	113	Fixed	0.153 (0.097–0.232)	−6.497	0.000						
Rash	4	88	Random	0.407 (0.210–0.639)	−0.782	0.434	3	48	Fixed	0.135 (0.074–0.234)	−5.423	0.000
Rash maculopapular	2	47	Fixed	0.101 (0.038–0.243)	−4.085	0.000						
Drug eruption	3	269	Random	0.111 (0.022–0.412)	−2.365	0.018

**Table 3:** Summary results of the all-grade and grade ≥ 3 adverse events (AEs) in combination therapies

adverse events	All-grade						Grade ≥ 3					
	No. of Studies	No. of Patients	model	Event rate with 95% CI	Z value	***p*** value	No. of Studies	No. of Patients	model	Event rate with 95% CI	Z value	***p*** value
Hematologic												
Anaemia	2	53	Random	0.687 (0.017–0.996)	0.319	0.750						
Lymphopenia	2	119	Random	0.619 (0.007–0.997)	0.176	0.861	2	119	Random	0.568 (0.004–0.998)	0.092	0.927
Thrombocytopenia	3	143	Random	0.248 (0.006–0.951)	−0.534	0.593						
Neutropenia	2	42	Random	0.812 (0.035–0.998)	0.600	0.549						
Gastrointestinal disorders												
Gastrointestinal disorder	2	53	Random	0.599 (0.001–0.999)	0.112	0.911						
Nausea	3	127	Random	0.159 (0.046–0.425)	−2.393	0.017						
Vomiting	3	127	Random	0.093 (0.032–0.242)	−3.945	0.000						
Diarrhea	2	103	Fixed	0.148 (0.091–0.231)	−6.240	0.000						
Stomatitis	3	143	Random	0.185 (0.035–0.585)	−1.592	0.111	2	119	Random	0.046 (0.004–0.393)	−2.290	0.022
General disorders												
Fatigue	3	127	Random	0.278 (0.072–0.659)	−1.159	0.246						
Pyrexia	3	143	Random	0.234 (0.017–0.844)	−0.810	0.418						
Chills	2	37	Fixed	0.110 (0.042–0.260)	− 3.930	0.000						
Oedema peripheral	2	37	Fixed	0.056 (0.014–0.200)	−3.863	0.000						
Injury, poisoning and procedural complications												
Infusion related reaction	2	37	Fixed	0.110 (0.042–0.260)	−3.930	0.000						
Metabolism and nutrition disorders /investigations												
AST increased	2	119	Random	0.163 (0.036–0.501)	−1.956	0.050	2	119	Fixed	0.059 (0.028–0.119	−7.103	0.000
ALT increased	2	119	Random	0.197 (0.030–0.663)	−1.325	0.185	2	119	Fixed	0.044 (0.019–0.102)	−6.696	0.000
Hyponatraemia	2	119	Random	0.060 (0.009–0.301)	−2.825	0.005	2	119	Random	0.032 (0.005–0.170)	−3.659	0.000
Hyperuricaemia	2	37	Fixed	0.056 (0.014–0.200)	−3.863	0.000						
Hypomagnesaemia	2	37	Fixed	0.056 (0.014–0.200)	−3.863	0.000						
Hypophosphataemia	2	53	Fixed	0.116 (0.053–0.236)	− 4.663	0.000						
Weight decreased	2	37	Fixed	0.113 (0.043–0.265)	− 3.878	0.000						
Platelet count decreased	2	103	Fixed	0.050 (0.021–0.114)	−6.436	0.000						
Decreased appetite	3	143	Random	0.275 (0.031–0.817)	−0.769	0.442						
Dehydration	2	37	Fixed	0.204 (0.099–0.373)	−3.166	0.002						
Hypotension	2	114	Fixed	0.021 (0.005–0.082)	−5.334	0.000						
Musculoskeletal and connective tissue disorders												
Arthralgia	2	37	Fixed	0.113 (0.043–0.265)	−3.878	0.000						
Musculoskeletal chest pain	2	37	Fixed	0.056 (0.014–0.200)	−3.863	0.000						
Myalgia	3	127	Fixed	0.041 (0.017–0.095)	−6.889	0.000						
Abdominal pain	2	37	Fixed	0.081 (0.026–0.223)	− 4.029	0.000						
Neck pain	2	37	Fixed	0.081 (0.026–0.223)	−4.029	0.000						
Back pain	2	37	Fixed	0.056 (0.014–0.200)	−3.863	0.000						
Chest pain	2	37	Fixed	0.056 (0.014–0.200)	− 3.863	0.000						
Skin and subcutaneous tissue disorders												
Rash	2	114	Fixed	0.370 (0.286–0.462)	−2.741	0.006						
Rash maculo-papular	2	114	Fixed	0.182 (0.121–0.266)	−6.046	0.000						
Skin exfoliation	2	114	Fixed	0.035 (0.013–0.090)	− 6.501	0.000						
Dry skin	2	114	Fixed	0.070 (0.036–0.135)	−7.033	0.000						
Infections and infestations												
Sepsis	2	37	Random	0.139 (0.017–0.607)	−1.584	0.113						
Pneumonia	2	53	Fixed	0.116 (0.053–0.236)	−4.663	0.000						
Pneumonitis	2	114	Fixed	0.021 (0.005–0.082)	−5.334	0.000						
Urinary tract infection	2	37	Fixed	0.056 (0.014–0.200)	−3.863	0.000						
Nervous system and psychiatric disorders												
Dysgeusia	2	114	Fixed	0.044 (0.018–0.101)	−6.738	0.000						
Headache	2	37	Fixed	0.142 (0.060–0.300)	−3.712	0.000						
Insomnia	2	37	Fixed	0.110 (0.042–0.260)	−3.930	0.000						
Malaise	2	114	Fixed	0.048 (0.020–0.110)	−6.509	0.000						

### Overall efficacy analysis

**Fig. 2:**
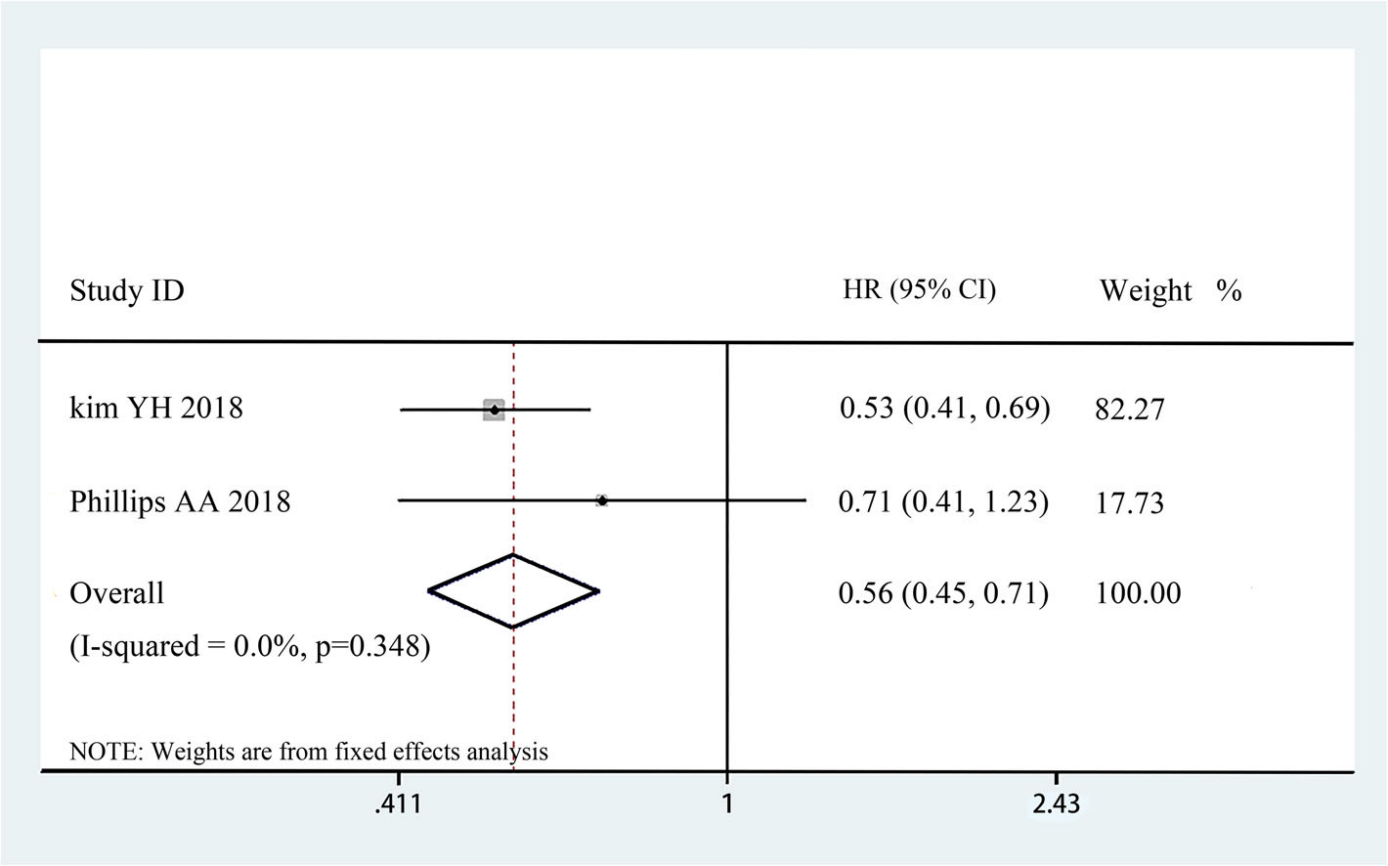
Analysis of the ORR of the studies in monotherapy and multiple therapies. (A) monotherapy; (B) multiple therapies

**Fig. 3:**
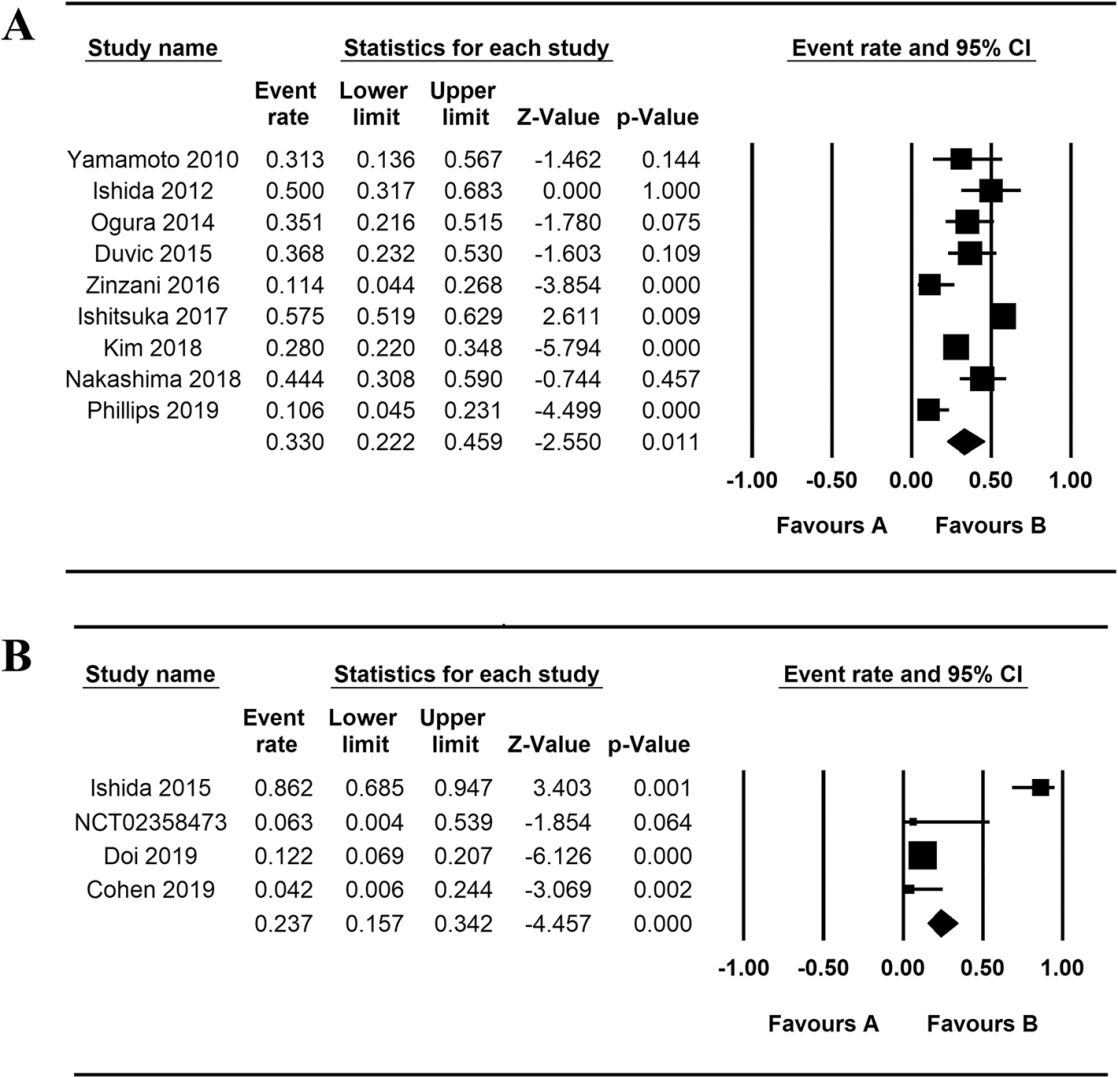
Overall analysis of the PFS of the studies in monotherapy。

**Fig. 4:**
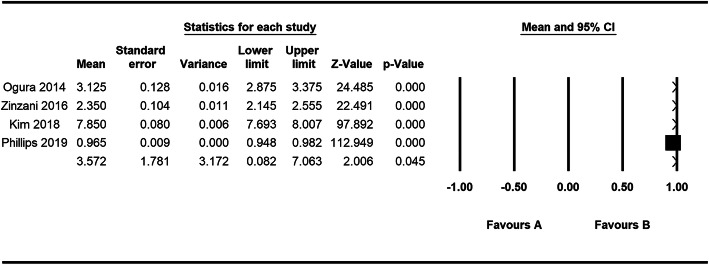
The HRs and 95% CI for PFS in control-arm trials

The pooled ORR rate, mean OS and mean PFS were used to measure the efficacy of mogamulizumab in treating cancers. For monotherapy, nine trials [14–17, 19–23] were included in the ORR analysis, and 4 articles [17, 20, 21, 23] were incorporated in the mean PFS analysis. According to our analysis, the pooled ORR rate was 0.430 (95% CI: 0.393–0.469) (Fig. 2A). The median PFS varied from 0.93 to 7.7 months and the pooled mean PFS was 1.060 months (95% CI: 1.043–1.077 Z = 125.452, *p* = 0.000) (Fig. 3). Besides, we performed further analyses to evaluate the efficacy between mogamulizumab and other chemotherapeutics. In controlled trials, the HRs for PFS in 2 control trials was 0.53 and 0.71 with a total HR of 0.56 (95% CI: 0.45–0.71, I-squared = 0.0%, *p* = 0.348) (Fig. 4), indicating a longer PFS in mogamulizumab group. For mogamulizumab in combination with other drugs, the pooled ORR rate was 0.203 (95% CI: 0.022–0.746) (Fig. 2B). Then we performed subgroup analyses to identify the pooled PFS and OS of patients with non-small cell lung cancer in combination therapies. The pooled PFS and OS were 2.435 months (95% CI: 1.752–3.119, Z = 6.982, *p* = 0.000) and 6.519 months (95% CI: 5.523–7.514, Z = 12.836, *p* = 0.000), respectively (Table 4).

**Table 4:** Subgroup analysis of PFS and OS in non-small cell lung cancer patients with multiple therapies

Study name	Year	Treatment	Cancer type	Media PFS (months)	Z-Value	*P*-Value	Media PFS (months)	Z-Value	*P*-Value
NCT02358473	2018	mogamulizumab +docetaxel	NSCLC	2.243 (95% CI:1.235–3.250)	4.364	0.000	7.335 (95% CI:5.657–9.013)	8.570	0.000
Doi, T	2019	mogamulizumab +nivolumab	NSCLC	2.600 (95% CI:1.669–3.531)	5.473	0.000	6.075 (95% CI:4.838–7.312)	9.629	0.000
Overall				**2.435 (95% CI:1.752–3.119)**	**6.982**	**0.000**	**6.519 (95% CI:5.523–7.514)**	**12.836**	**0.000**

*Abbreviation*: *NSCLC* non-small cell lung cancer

### Assessment of study quality and publication bias

**Fig. 5:**
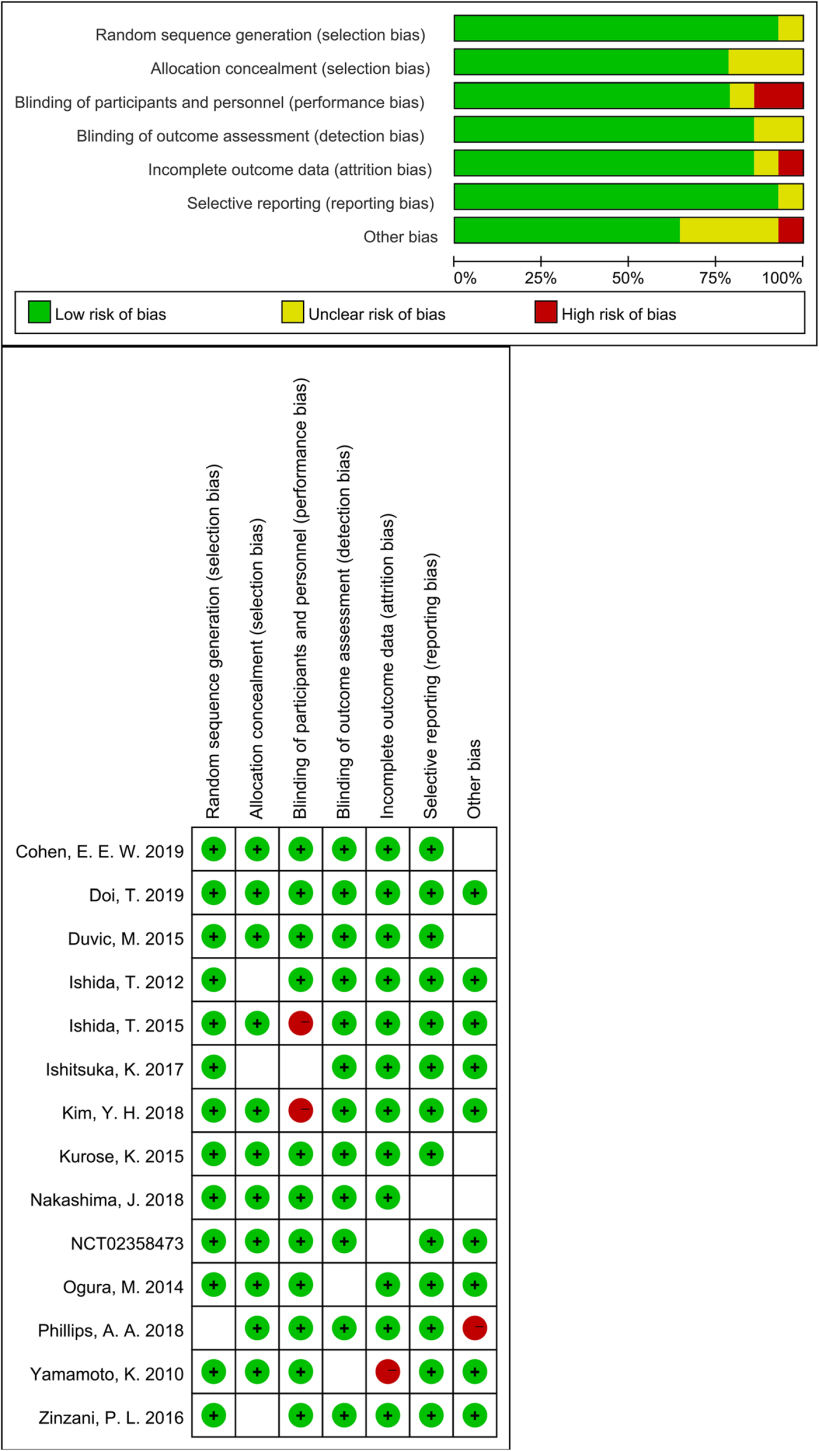
The risk of bias graph and the risk of bias summary

We used Review Manager 5.3 (Copenhagen, Sweden) to measure quality assessment of involved studies. Figure 5 indicated the risk of bias graph and risk of bias summary of all those eligible trials. Overall, the quality of the studies was satisfactory.

## Discussion

Although various advanced or metastatic malignancies remain incurable, the application of mogamulizumab does benefit the patients. In this meta-analysis, we selected 14 prospective trials with 1290 patients and systematically assess the safety and efficacy of mogamulizumab. The integrated results of the data analysis confirm the role of mogamulizumab in various cancers. This is the first time to evaluated the safety and efficacy of mogamulizumab independently and systematically. This meta-analysis reveals that mogamulizumab is a novel therapy in treating various cancers, offering powerful evidence for clinical decision.

With regard to the safety, we analyzed the AEs of patients administered mogamulizumab by intravenous infusion. According to our analysis, the most common all-grade AEs in low, medium and high doses were lymphopenia, and event rates of lymphopenia were all higher than 70%. Besides, lymphopenia was also the most common grade ≥ 3 AEs which happened in nearly half of the participants. For combination therapies, neutropenia was the most common AEs which happened in more than 80% patients while lymphopenia was the most common grade ≥ 3 AEs which happened in nearly half of the participants. Most of the AEs associated with mogamulizumab were mild and reversible. The observed lymphopenia in all doses and all mogamulizumab-related therapies was considered the pharmacologic effect of mogamulizumab. Though transient, infusion reaction was the most common nonhematologic AEs. The mogamulizumab, having a defucosylated Fc region, has increased binding affinity to the Fcγ receptor on effector cells such as NK cells which can enhance ADCC. By strongly activating NK cells, mogamulizumab can induce NK cells to release cytokines and related cytotoxic molecules which might be the mechanism of infusion reaction [15, 28, 29]. Mogamulizumab can reduce the level of CCR4-positive malignant T cells locally and systematically, and can also eliminate CCR4-positive Tregs leading to Tregs depletion, which contributes to the enhancement of antitumor effects and the immunotherapeutic effect of activating the host immune response [18, 30]. However, mogamulizumab induced Tregs depletion can cause alteration of the immune balance, which may unleash various undesirable infections [22, 31]. Skin-related AEs were another frequent nonhematologic AEs because CCR4 can promote skin-specific homing of lymphocytes, while mogamulizumab was a monoclonal antibody directed against CCR4 [32, 33]. Mogamulizumab induced Tregs depletion may abrogate a peripheral checkpoint which is controlled by Tregs and induce the production of autoantibodies. These autoantibodies can combine with keratinocytes and melanocytes which play an essential role in the pathogenesis of skin-related AEs [34, 35]. In addition, some studies revealed the mogamulizumab treatment could provoke homeostatic CD8-positive T-cell proliferation predominantly of newly emerging clones, some of which may also play an important role in the pathogenesis of skin-related AEs [35–37]. For combination therapies, most of the AEs showed similar trends to those in monotherapy which was consist with previous studies. In combination therapy of mogamulizumab and nivolumab treating solid tumors, the profile of AEs was not substantially different from that seen in mogamulizumab or nivolumab monotherapy [25].

With regard to the efficacy, we analyzed the ORR, PFS, OS and HR for PFS of included trials. Approximately 43% of participants reached complete response or partial response in monotherapy. This is a particularly promising result since the response rate of patients with ATL, PTCL, CTCL ect, to conventional chemotherapy with a single agent is reportedly extremely low. The mogamulizumab is a humanized, IgG1 kappa monoclonal antibody directed against CCR4 which can highly enhance antitumor effects by reducing the number of CCR4-positive leukemic cells and Tregs leading to Tregs depletion [38]. The mechanism of the decline of CCR4-positive leukemic cells is that mogamulizumab has increased binding affinity to the Fc receptor on effector cells such as NK cells which can enhance ADCC [29, 39]. The mechanism of Tregs depletion is that mogamulizumab prevents the binding of CCR4 and CCL22 so that the Tregs cannot be activated and recruited [38]. So the mechanism of antitumor activity is that the mogamulizumab can reduce the number of CCR4-positive leukemic cells, boost antitumor immunity by reducing CCR4-positive Tregs and influence tumor microenvironment to reduce tumor escape [40]. Patients administered mogamulizumab stabilized the disease more than 1 month and the overall patient survival time ranged from 4.9 months to 17.6 months which were longer than existing standard therapies. The analysis of HR for PFS in eligible trials also indicated that mogamulizumab can prolong the PFS of cancer patients compared to other chemotherapeutics. In the combination therapy of mogamulizumab and mLSG15 treating ATL, the pooled ORR was higher than that of mogamulizumab monotherapy, which was related to the different mechanism of antitumor effects. However, in combination therapy of mogamulizumab and nivolumab or utomilumab treating solid tumors, the pooled ORR was relatively low, which was related to the characteristics of the treated tumors. But mogamulizumab combined with nivolumab or utomilumab were more effective than nivolumab or utomilumab alone in treating solid tumors [24, 25, 41]. These results above demonstrated that mogamulizumab is a novel therapy in treating patients with cancers either as a single drug or in combination with other drugs.

However, there are several limitations to this study. Firstly, the data of this meta-analysis is limited and some included studies even miss partial data. More experiments with larger sample size and more comparisons with other drugs are required. Second, the deficiencies in the experimental design of the selective studies cannot be eliminated from our analysis. Third, some patients might receive different prior systemic chemotherapy regimens, which could affect the results of the present study. Besides, there are diversities in the study design of different experiments such as experiment duration. Finally, the cancer types are different, so the heterogeneity of the enrolled patients might have affected the results.

## Conclusions

Based on the current evidence, this meta-analysis elucidates that mogamulizumab has clinically meaningful antitumor activity in patients with an acceptable toxicity profile which is a novel therapy in treating patients with cancers.

## Data Availability

All data are available in this manuscript.

## Abbreviations

CCR4: CC chemokine receptor 4
ATL: Adult T-cell leukemia/lymphoma
PTCL: Peripheral T-cell lymphoma
CTCL: Cutaneous T-cell lymphoma
OS: Overall survival
PFS: Progression-free survival
ORR: Objective responses rate
HR: Hazard Ratio
AEs: Adverse events
CI: Confidence interval
ADCC: Antibody-dependent cellular cytotoxicity
PRISMA: Preferred Reporting Items for Systematic reviews and Meta-Analyses
CTCAE: Common Terminology Criteria for Adverse Events
RCT: Randomized controlled trial
